# Facilitating Perinatal Access to Resources and Support (PeARS): a feasibility study with external pilot of a novel intervention

**DOI:** 10.1186/s12884-021-04112-w

**Published:** 2021-11-12

**Authors:** Pauline Slade, Melanie Dembinsky, Katie Bristow, Kim Garthwaite, Amy Mahdi, Annette James, Atif Rahman, Soo Downe

**Affiliations:** 1 Institute of Population Health, University of Liverpool, Ground Floor Whelan Building, Brownlow Hill, Liverpool, L69 3GB UK; 2 Health Sciences Stirling, University of Stirling, Stirling, FK9 4LA UK; 3 NIHR CLAHRC North West Coast, Department of Health Services Research, Institute of Psychology, Health and Society, University of Liverpool, Waterhouse Building, Brownlow street, Liverpool, L69 3GL UK; 4 Liverpool City Council, 1 Municipal Buildings, Dale St, Liverpool, l2 2DH UK; 5 Liverpool Women’s Hospital NHS Foundation Trust, Crown St, Liverpool, L8 7SS UK; 6 Head of Children’s Health Improvement, Liverpool Public Health, Liverpool City Council Cunard Building, Water Street, Liverpool, L3 1DS UK; 7 Institute of Population Health, University of Liverpool, Waterhouse Building, Brownlow street, Liverpool, L69 3GL UK; 8 School of Community Health and Midwifery, University of Central Lancashire, Preston, PR1 2HE UK

**Keywords:** Pregnancy, Postnatal, Mental health, Community services, Peer, Implementation intentions

## Abstract

**Background:**

Up to 50% of women in areas of high socio-economic deprivation are at risk of developing depressive symptoms in pregnancy. Feeling well supported, can facilitate good mental health perinatally. A brief, innovative intervention to facilitate access to support and resources was developed and tested. This included one antenatal and one postnatal session, each with three evidence-based components: i) support from a non-professional peer to enable a woman to identify her needs; ii) information about local community services and signposting; and iii) development of a personalised *If–Then* plan to access that support. The aims were to evaluate the intervention and research methods for feasibility and acceptability for perinatal women, maternity care providers and peers, and provide preliminary effectiveness indications.

**Methods:**

Pregnant women living in an area of high deprivation were recruited from community-based antenatal clinics and randomised to intervention or control condition (a booklet about local resources). Outcome measures included women’s use of community services by 34 + weeks gestation and 6 months postnatally; mental health and wellbeing measures, and plan implementation. Interviews and focus groups were conducted with women participants, providers, and peers. Data were analysed using framework analysis. Recruitment and retention of peers and participants, intervention fidelity, and acceptability of outcome measures were recorded.

**Results:**

Peer facilitators could be recruited, trained, retained and provide the intervention with fidelity. One hundred twenty six women were recruited and randomised, 85% lived in the 1% most deprived UK areas. Recruitment constituted 39% of those eligible, improving to 54% after midwifery liaison. Sixty five percent were retained at 6 months postnatally. Women welcomed the intervention, and found it helpful to plan access to community services. Providers strongly supported the intervention philosophy and integrated this easily into services. The study was not powered to detect significant group differences but there were positive trends in community service use, particularly postnatally. No differences were evident in mental health and wellbeing.

**Conclusions:**

This intervention was well received and easily integrated into existing services. Women living in highly deprived areas could be recruited, randomised and retained. Measures were acceptable. Peer facilitators were successfully trained and retained. Full effectiveness studies are warranted.

**Supplementary Information:**

The online version contains supplementary material available at 10.1186/s12884-021-04112-w.

## Background

Almost half of pregnant women in areas of high deprivation screen positive for perinatal depressive symptoms [1, 2]. Feeling well supported during the transition to parenthood promotes maternal wellbeing [3]. The value of support provided by peers has been demonstrated to be effective in reducing perinatal depression [4]. Improved maternal wellbeing and mental health is important as it impacts not only on the woman [5] but also on the growing fetus and other outcomes [6, 7].

Research has highlighted several breaks in the perinatal mental health care pathway leading to low uptake of available resources in socio-economically disadvantaged women [8, 9]. Access and uptake of available support may occur least where the need is greatest. Barriers to uptake include stigmatization of mental health issues [10]. A bridge between need and available resources may have the potential to facilitate the mental health and wellbeing of women in areas of high social deprivation.

To address the gaps between need and uptake of resources a low-cost, brief (20 min) peer-delivered intervention was developed from three evidence-based elements which, separately, have previously been shown to increase access to local community services and psychosocial wellbeing in other contexts:

- 1. Peer facilitators specially trained with a supportive non-judgemental approach to help women identify their own needs
- 2. Use of an interactive community map including local resources, opening times, and transport links to enable signposting
- 3. Action-planning (‘If–Then’ planning) to translate intentions to use services into a personal action plan.

These three integrated elements are derived respectively from Rahman’s global work on peer-delivered mental health interventions [11, 12]; the community engagement components from the Access to Mental Health in Primary Care (AMP) study in North-West England [13] which used a signposting element via the use of a specifically constructed community map of existing resources; and, finally Slade’s ‘*If–Then’* planning [14, 15]. This is based upon the principle proposed by Gollwitzer [16] that developing an implementation intention, by planning the ‘what, when and how’ of a behaviour, can bridge the intention behaviour gap i.e. the gap between what people intend to do, and what they actually do [17]. These three elements were integrated and shaped for the perinatal context into a novel intervention.

The three elements were chosen to address some crucial barriers to supporting mental health during a time of change and strain. A peer delivered intervention can reduce stigma which is recognized as an important barrier to accessing services and facilities [10] signposting to local resources provides important informational and potentially emotional support; [18] and If–Then action planning has been shown to be effective in enabling people to put their intentions into action across a range of health domains [17]. It is particularly useful in contexts where attention, memory and self control may be low, such as in already high stressful life circumstances. This is because If-then planning  facilitates recognizing opportunities for action which becomes more automatic and requires less conscious processing [19].

Whilst other elements could be equally relevant, an intervention with a combination of these features in the context of a warm, non-judgemental approach from peer supporters, was felt to have potential utility. In addition implementation intentions developed collaboratively with another person have been shown to be more effective than when developed alone [20]. The study was also based on a timely expert consensus report identifying optimal ways for utilising implementation intentions [21].

The intervention was conceived as a multiagency partnership between statutory United Kingdom National Health Service maternity service providers, with midwives facilitating recruitment of women in their care, and non-statutory organisations (third sector agency, Children Centres) who then employed peers to be trained. Supervision of intervention delivery was provided from within the non-statutory agencies and day-to-day governance was provided by the research team. Full details of the development of the Perinatal Access to Resources and Support Intervention (PeARS) are available in the full report [22].

### Aims

This study aimed to assess the feasibility and acceptability of the PeARS intervention, and research process for perinatal women, community maternity care providers (midwives and health visitors) and peer facilitators. This included assessing the possibility of training and retaining peer facilitators to deliver the intervention with fidelity, and the recruitment of women living in areas of high deprivation to a randomised study of this intervention and to provide preliminary indications of effectiveness.

## Methods

### Development of the community map

The process for this is fully outlined in the PeARS report [22]. In summary, this identified all local community facilities, together with information on timings about availability. These were grouped under one of eight categories (Table 1) identified as important by preliminary local qualitative work with perinatal service users. They were incorporated into an interactive community map that included transport options and contact details for services. As community resources can frequently change, and accuracy of information in the community map was paramount, it was updated on a three monthly rotation by an administrator from non-statutory services.

**Table 1:** Womens needs and exemplars of community services and resources

*What if I want to?*	Type of service/ resource	Example of service/ resource
Improve my health, wellbeing and fitness	Local Health & Fitness services	MAMA fit
Meet new people and take part in local activities	Local Leisure activities	Everton in the Community
Learn about and prepare for being a new mum (antenatally) / *Learn about activities for me & baby( postnatally)*	Local pregnancy/postnatal support	Antenatal classes Pregnancy massage //Postnatal groups
Talk about how 1’m feeling	Local and national wellbeing and support services	Relate Cheshire & Merseyside/ Family Helpline
Seek advice & support	Local Advice with housing, benefits, etc	Citizen’s Advice Bureau
Learn something new or find a job	Local Adult learning	Community Centre and local College courses
Speak to a health professional	General Practitioners/Midwives	Local Health centres
Find breast feeding friendly places (postnatal only)	Local directories	

### Recruitment of peer facilitators

Ten peer facilitators and three peer facilitator supervisors were recruited from participating Children’s Centres^1^ and local voluntary organisations. In order to be considered a peer for this study, each individual needed to have a) no formal training as a health or social care professional; b) personal experience of pregnancy and motherhood; c) no additional involvement with the study participants in any other capacity; d) evidence of a warm and approachable personality; and e) to be living locally.

### Training of peer facilitators

Peer facilitators and peer facilitator supervisors attended a four short day training course specifically developed by the research team for this project. The peers were trained to provide an exploratory and non-directive discussion relating to what sort of support or activities the women themselves thought would be of most value to them. The approach was based on the principles of warmth, empathy and genuineness coupled with the list of eight categories identified by the a priori qualitative work, and the community map. The intention was to find the best fit for each individual service user, and then to work collaboratively with them to develop an implementation plan.

The core components were therefore as follows: the development of engagement, exploratory questioning and empathic listening skills to enable identification of women’s views on their needs; familiarisation and successful utilisation of the interactive community map of local community resources and transport links to enable individualised sign-posting to accessible community based resources/services; and to facilitate *If–Then* action-planning to the resource best meeting participant needs and preferences.

Training briefly addressed the basic psychological theory underpinning implementation intentions, and identified the key components of action planning, collaborative development of a personal plan via *where, when* and *how* components. In addition, identification of barriers to action and exploration of ways of overcoming barriers i.e. the development of a COPING ‘*If–Then’* plan was completed. Peers were trained to support each woman to write, text or voice record their own personal plan.

An example of a plan for a woman interested in improving health and wellbeing might be:

*‘IF I have just returned home tomorrow morning after dropping my daughter at nursery THEN I will phone the MAMA fit class to book sessions for Wednesday PM at (…. location).’* A coping plan might identify a potential barrier such as that class being full. An example of a coping plan might be.*.’IF that is not available THEN I will ask my mother if she will look after my daughter so I can attend an evening session*’. Peers were trained to make a follow-up phone call two weeks after the intervention, to enquire how each woman ‘had got on with her plan’, or if this needed amendment in any way. To guide them in situ peers were trained to use a checklist of steps and noted plan content.

The success of the training for each peer facilitator was measured by an observer who rated their end of training intervention role plays, against a pre-determined set of criteria. The criteria were focused on content and process. All the elements of the intervention needed to have been included for the maximum fidelity score to be awarded ( e.g. identification of a need; linking to a resource related to that need; and the development and rehearsal of a plan to address the need. The interaction with the woman also had to be provided in an exploratory, warm, and supportive manner). In effect the intervention was divided into 13 observed content steps (what was done) and 4 process aspects (the way it was undertaken). These consequent training criteria were later used as the fidelity monitoring checklist. A booster training session for all peer facilitators was held, to refresh their training between the antenatal and postnatal peer facilitations. During these training sessions all aspects of the role were addressed to adequately prepare potential peer facilitators for their new role. Structured one hour group supervision was then provided for peers on a 4–6 weekly basis by supervisors, trained in the approach and supported by the researchers. Throughout the study intervention fidelity monitoring took place,and 10% of actual intervention sessions were rated as present or absent by a researcher using the fidelity monitoring checklist (see Additional file 2).

### Study design

This was a feasibility study with an external pilot. The main purpose was to provide qualitative process information from participants and staff. Qualitative data were collected from women participants 6 months postnatally and from maternity care providers, peer facilitators, and peer facilitator supervisors.

Quantitative measures were collected at baseline; TIME 1—booking visit in early pregnancy; TIME 2—34–37 weeks gestation (antenatal follow-up); and TIME 3—6 months postnatally (postnatal follow-up). The primary quantitative outcome was community service use. Secondary outcomes included psychosocial wellbeing measures.

### Participants and recruitment

The study was carried out in six wards in a Northwest UK city chosen as representative of areas of high socio-economic deprivation. Participants were recruited from six Children’s Centres that provided antenatal community-based midwife booking-in clinics (first contact with a midwife in pregnancy). All service user participants were pregnant women, registered with local GP practices and attending these booking clinics.

The inclusion criteria were: over 18 years of age, under community midwifery led care and proficient in the English language. Women were excluded if they were already being provided additional care by the Family Nurse Partnership, the perinatal mental health team or the enhanced midwifery team (for drug or alcohol misuse; domestic violence or safeguarding issues). They were also excluded if they planned to move out of the study area within six months. Women requiring translation facilities routinely received their booking appointments at the central hospital not the community clinic and were not included.

Information sheets about the study were provided with the booking appointment letter. At the booking visit the midwives checked eligibility; those meeting the inclusion/exclusion criteria were invited to meet a researcher. If the woman consented to participate, baseline demographic and mental health measures, as indicated below, were completed.

Randomisation to intervention or control was stratified by parity, and automated using the Sealed Envelope simple randomisation service. Initially, a 1:1 randomisation system was used. This was later changed to a 1:2 scheme, in favour of the intervention group, to enable peer facilitators to build skills and experience in the provision of the intervention, and, therefore, to provide enhanced evidence about feasibility and acceptability of the intervention once peer supporters were confident in their role. All women received the usual ongoing maternity care.

Women randomised to the intervention group immediately met with a peer facilitator who provided the antenatal element of the intervention. The peer facilitator contacted each woman by phone approximately two weeks after the first visit, to see how she had got on with her plan, or if she needed to change this. Women randomised to the control group were given a booklet by the researcher about their local resources. Postnatally intervention group women were offered a further meeting at about 6 weeks after birth with the same peer facilitator and same components of support: identification of needs, tailored postnatally and signposting through use of the community resource map and development of an ‘*If–Then’* plan with a 2 week follow up phone call. Research assessments of community resource use and mental health were completed by phone/post/online at 34–37 weeks gestation (TIME 2), and repeated at 6 months postnatally together with measures of parenting stress and feelings for the infant (TIME 3).

### Measures

At TIME1 Baseline levels of anxiety and depressive symptoms and wellbeing using the Hospital Anxiety and Depression Scale (HADS) [23] and the Warwick Edinburgh Mental Wellbeing Scale (WEMWBS) [24] were completed.

At TIME 2 (34–37 weeks gestation) the HADS and WEMWBS were repeated and the main outcome, the uptake of community services through self-report on the service utilisation section of the Client Service Receipt Inventory (CSRI from booking to pregnancy follow up), recorded [25]. The dimension of particular interest was use of community services, which was recorded in terms of number and duration of contacts.

At TIME 3 (6 months postnatal) HADS, WEMWBS, were repeated. The CSRI was repeated to capture community contacts from 6 weeks to 6 months postnatally. In addition the Mother Object Relations Scale (MORS) [26] and the Parenting Stress Index (PSI-SF) were administered [27]. The latter tool measures parental distress (PD), perceived difficult child (DC) and parent–child dysfunctional interaction (PCDI).

### Interviews and focus groups

To assess women’s perspectives consequent on their randomisation to either the intervention or the control group, all participants were asked at TIME 3 if they wished to complete a telephone interview about their experiences of participating in the study, and their experiences of the intervention or for the control group their views on the described intervention. Semi-structured telephone interviews with 49 (26 intervention and 23 control) women were completed. Topic Guides were developed for these and are included as Additional file 1.

For staff perspectives 5 focus groups and 5 interviews with midwives (N = 9), health visitors (N = 6) and peer facilitators (N = 8) and 3 peer facilitator supervisors involved in the study were conducted.

The interviews and focus groups covered their experience of involvement in the intervention and their views on all aspects of the intervention, benefits and obstacles in practice. Focus groups and interviews were audio recorded and transcribed verbatim. NVivo software package was used to aid in data management and analysis.

### Quantitative data analysis

Data were analysed descriptively and through group comparisons, repeated measures and analysis of variance across time points.

### Qualitative data analysis

Data were initially coded separately by two independent researchers using a thematic framework analysis approach [28]. This retains links to the raw data after reducing it through summarisation and synthesis. After the initial coding, study researchers (MD, KB and PS) discussed and agreed emergent themes and subthemes. Disconfirming information was specifically sought. In line with the underpinning collaborative philosophy of the work as embedded with stakeholder communities the preliminary themes and subthemes and their evidence were then presented, and discussed at two separate stakeholder analysis workshops covering staff and women participant perspectives respectively. These workshops brought together stakeholders from a variety of disciplinary backgrounds and service users with a shared interest in perinatal (mental) health and service provision to review the data and preliminary analysis. Through discussions of the evidence over the course of the workshop, emerging themes and subthemes were further refined and agreed, making the analysis process more rigorous and transparent. Initially, midwives, health visitor and peer facilitator focus groups were analysed separately. However, it became apparent that there was strong synergy across staff and peer groups and an overarching analysis is presented.

## Results

These are structured to present quantitative information about peer facilitators, and women’s recruitment, retention and plan implementation, then process information about women’s, staff and peer perspectives and finally preliminary data about effectiveness.

### Quantitative findings

#### Recruiting, training and retaining peer facilitators and fidelity checks

Eight of 10 peer facilitators were retained throughout the 18-month study period. Two left due to changes in employment circumstances. One supervisor left her post. None of the losses were due to the peer role: in each case, the individual experienced changes in their primary employment. There was no difficulty recruiting and retaining peer facilitators.

Fidelity checks on the intervention provision indicated that trained peer facilitators could reliably deliver the intervention. Fidelity monitoring indicated from ten observed interventions and therefore 130 elements (i.e.10 × 13 elements per intervention) only three elements were absent or inadequately delivered and formative feedback was provided.

#### Recruitment and retention of women from areas of high deprivation

One hundred twenty six (46%) of 297 eligible women invited agreed to take part. Sixty-eight were randomised to peer facilitation and 58 to control. After liaison with midwives and development of a clear recruitment protocol, the recruitment rate in the latter half of the study rose to 54%. The mean age of the recruited sample was 28 years. Forty two percent of participants were experiencing their first pregnancy, 81% were married or in a relationship, 56% were in employment and 83% identified as white British. Eighty five percent of the sample lived in the most deprived 10% of the UK according to the Index of Multiple Deprivation (IMD) scores. Women randomised to intervention and control groups did not differ on demographic or baseline (TIME 1) mental health measures.

After exclusions for miscarriage and referral to enhanced midwifery post randomisation, 101 women remained eligible to continue to TIME 2 (34 + weeks of pregnancy). Fifty-nine women (58%) completed the antenatal study measures at TIME 2 and 66 (65%) completed the postnatal measures at TIME 3 (6 months postnatally). Forty-eight women completed all three time points.

Those who completed follow up at TIME 2 were significantly older (M = 29.4 years, SD = 5.10) than those who did not (M = 27.43, SD = 5.52, t = -2.01 *p* = 0.047). Similarly, TIME 3 follow up completers were significantly older (M = 29.3 years, SD = 5.00) than non-completers (M = 26.8 years, SD = 4.42, t = -2.48, *p* = 0.014). There were no other differences in terms of demographic variables or baseline psychological wellbeing measures between follow-up completers and non-completers at TIME 2 or TIME 3.

#### Plan implementation

Of the 52 intervention group participants who were still eligible for follow up at TIME 2, 26 (50%) reported that they had completed their antenatal ‘*If–Then’* plan. Eight (15%) women reported that they had been unable to complete this and 18 were recorded as missing data. Half (50%) of antenatal plan completers attended postnatal peer facilitation compared to 23% of women who did not complete their plan or for whom there was no information. Only 19/52 (37%) accepted the postnatal invitation and attended for postnatal peer facilitation, but 18/19 (95%) of these women successfully completed their postnatal ‘*If–Then’* plan.

### Process findings

#### Women’s perspectives

One overarching theme and four themes each with constituent subthemes were identified for the participants’ interview data (Table 2). *Bringing Mum into Focus* was identified as an overarching theme. This expressed the way participants felt that the intervention was individually tailored, permitting women to express their own personal needs in the context of the perinatal phase, and legitimising their rights to have these needs met. The four themes covered: *experiences of the interventions*, *how when and for whom the intervention should be provided, perspectives on research participation* and *use of the resource booklet.* Intervention women are indicated by I and Control by C and a participant code number.

- 1. Experiences of the intervention
  “I think it’s useful” (NOTE the intervention provided) (I 42)
  The first subtheme, *Enduring memories of benefits of a positive experience,* reflects women’s overwhelming views that the intervention was acceptable and was felt useful. These were in relation to the intervention as a whole, and included the identification of needs, matching this with accessible resources and being supported to devise a plan of action with a warm supportive peer.

“I think that would be brilliant” (NOTE the provision of the described intervention) (C 3)

**Table 2:** Women’s perspectives: themes and subthemes

Theme	Subthemes
1. Experiences of the intervention	1. Enduring memories of benefits of a positive experience Antenatal a. From useful to brilliant b. Route to information c. Receiving support d. Being known 2. Things that made it not so useful a. Experiencing it as hard or unrealistic or hard to process at the time as overwhelmed by pregnancy or feeling unwell b. Not attending postnatal peer facilitation 3. What got in the way of implementing their plan a. Services not available b. Other commitments c. Pregnancy related illness 4. Relationship with peer facilitator a. Warmth b. Efficiency c. Continuity of person and feeling known
2. How when and for whom the intervention should be provided	1. Intervention timing a. Antenatal -Early pregnancy b. Postnatal- After 6 weeks 2. Who should be offered the intervention a. Universal with self-determined access 3. How should it be organized a. Fine the way it was b. Other suggestions c. Lay person should conduct intervention
3. Research study participation	1. Motivations for participating a. Perceiving own vulnerability b. Wanting to help c. Wanting information 2. Experience of the study a. Not recalling all information processes but this was fine b. Able to ask all questions needed c. Completing baseline questionnaire was fine 3. Attitudes toward being involved in the study a. Being involved was fine b. Too intrusive
4.Use of resource booklet	1. Looked at it 2. Looked at it & used it 3. Didn’t look at it or use it
**Overarching theme: *Bringing Mum into focus***	

Women saw this as a route to information and support and ‘feeling known’.

Subtheme 2 covered the few women who indicated that there were Things that made it not so useful. These were, not being able to focus on the peer facilitation meeting during the initial antenatal session due to feeling overwhelmed by the confirmation of the pregnancy, or because a woman was not feeling well in the pregnancy or if they were experiencing their personal plan as hard or unrealistic.

A third subtheme was Things that got in the way of implementing their plan. Women were asked whether or not they were able to implement their ‘If–Then’ plan. Where women indicated that they had not, a follow-up question explored the reasons why. Reasons given ranged from suffering from severe pregnancy-related illness or services not being available on a suitable day to struggling finding the time to do things for self. The latter included having other commitments such as work or other children, this was also the most frequently stated potential barrier anticipated by control group women. Other suggestions were more along the lines of practical problems such as access to parking and public transport to specific activities included in their plan:

“Parking and just obviously when you are not feeling great and getting out and having to get a bus or a train or whatever … I think a cost if there was a cost something like that would be a factor” (I45).

The fourth and final subtheme under this major theme was Relationship with the peer facilitator. All women commented positively on this relationship, noting, for instance, the warmth and efficiency of the peer facilitators. This illustrated how much women valued their interactions despite the intervention consisting of only two brief face-to-face meetings. Women repeatedly expressed the desire for continuity of carer, and were delighted this continuity was reflected in the structure of the intervention which enabled them to feel comfortable, known, heard and respected.

“I think that’s [having the same peer facilitator for antenatal and postnatal meetings] better because that person might know about you, how you are emotionally and after the baby they can see the difference or … rather than information just getting passed out from person to person, so it should be the same person” (I 41).

- 2. How, when and for whom the intervention should be provided
  “It has to be available for every woman who needs it.” (I9)
  The second theme related to the views of intervention and control group women about how the intervention was best provided. Subthemes included the best timing, who should be able to access this intervention and how it was best organized. All women agreed that if this model of support were to be available in future, it should be offered early in pregnancy and to all pregnant women.

The majority of women also felt that current organisation of the scheme was effective, and that the intervention itself should be delivered by a nonprofessional i.e. a lay person. This raised issues of mistrust of health care professionals among this population group.

“I think a lay person be best, because I think when it's kind of a health professional, people can feel quite judged … you know the kind ow, I'm being checked up on, … I know she's [peer facilitator] obviously got a duty of care and responsibilities … but it's not as kind of intrusive in that way” (I49).

The majority of women indicated that the way the intervention was provided was broadly acceptable to them, with some caveats. Women emphasised that they preferred the meetings to be combined with an already standing appointment (especially during the antenatal period), such as their initial antenatal booking-in appointment with the maternity services. They felt that an additional stand-alone appointment would be too much on top of the multiple appointments women already have during pregnancy. They thought postnatal peer facilitation meetings should be offered later than provided in the study, and not before 8 weeks postnatally with the option of having the peer facilitation delivered as a phone consultation.

“Because people like.. they all have busy lives … that would be a lot easier you could just pick up the phone instead” (I11).

- 3. Research study participation
  “It was fine, yeah. It was quite straight forward” (I16)
  This theme included the subthemes of motivations for participation in the study, experience of participation in the processes and procedures of the research and overall attitudes about participation.
  Women were motivated by their own sense of need and vulnerability but also to help others in the future. With reference to the process of taking part this was seen as unproblematic:

The process for the research had been that women booking in for their initial midwifery appointment at any of the participating Children’s Centres received a letter, in addition to their booking-in appointment letter, explaining that the study was being conducted at the location they were booking in, and providing details. When asked how they had experienced recruitment to the study the majority of women were unable to recall the additional letter but reported being unconcerned by whether they had accessed information about the study before their appointment.

“No, I don't think so … no. I don't think it would have made a difference to be honest” (I49)

This was mitigated by the fact that women stated that they were able to ask any questions they had, and these were answered satisfactorily.

“Yeah … the questions that I did have were all answered thoroughly” (I5)

Women found the measures of outcome acceptable and welcomed the different (phone, postal, online) options to complete and return the measures. Overall research participation was experienced positively except for one woman who reported that ‘it (being in the study) was too intrusive’.

- 4. Use of Resource Booklet
  The final theme concerned use of the resource booklet for the control group. When asked about their use of this booklet, most women in the control group stated that they had not used it aside from a cursory glance. This suggests that simply providing information in leaflet/booklet form alone is not enough to engage perinatal women or to enable them to access local services.

#### Staff perspectives

Five themes and one overarching theme (Table 3) were identified from the staff interviews and focus group data. *Going where women are* is the overarching theme that highlights staff perceptions of the intervention’s flexibility in timing and place, how services are delivered, and the individualised care principle it is based on. It captures both practical convenience and an emotional engagement. This was said to be particularly pertinent by maternity care providers, as, in their day to day service provision, they faced high levels of mistrust from the women in the study area.

- 1. Feasible to integrate within existing services and practices
  “I think as well, that you know, it's an important piece of work that fits in very well with what we do as a service” (PF2).
  Perspectives were from midwives directly involved with the study through recruitment and detailed knowledge of the intervention design, and health visitors who were not directly involved in study process but had received a detailed briefing of the intervention. The first theme identified from both focus groups was that it was Acceptable, desirable, and feasible to integrate the intervention within routine maternity care services. Midwives and health visitors showed great enthusiasm for the intervention and stated that they did not anticipate any difficulties integrating this into their routine practice. Indeed, health visitors felt that they themselves were well positioned to talk about the intervention, or even deliver it themselves.
  Peer facilitators said that providing the intervention was rewarding and easily integrated within their existing roles at the Children’s Centres or employed in third sector organisations. One respondent reported that the peer facilitator role aligned strongly with their organisation’s philosophy:
- 2. Intervention implementation issues
  “That was a bit frustrating because you couldn’t do anything about it, you knew that this trial was in place, there could be potential benefits for them and we couldn’t they just didn’t fit the criteria” (MW3).
  A subtheme for midwives and peer facilitators was about the best timing of the first session of the intervention after what was a time-consuming antenatal booking-in appointment (typically 60–90 min). It was acknowledged that creating an extra session would bring its own set of problems. After some discussion within the focus groups which was mirrored in the stakeholder group, it was agreed that, despite the additional time and information load, the booking-in appointment is logistically the easiest and most reliable time with regards to women’s attendance. In addition, there were issues about who should receive the support package, with midwives preferring to go beyond the study inclusion criteria, to offer ALL women the intervention in future.
- 3. Managing expectations and emotions
  A third theme concerned midwives’ frustrations on managing exclusions.

**Table 3:** Staff and Peer Perspective Analysis Themes and subthemes across staff midwives (MW) and health visitor (HV) and peer facilitator (PF) groups

Theme	Subtheme
1.Feasible to integrate within existing services and practices (MW HV PF)	● Acceptability ● Added value ● Conducting the intervention
2.Intervention implementation issues (MW HV PF)	● Intervention timing in pregnancy ● Who should be eligible
3. Managing expectations and emotions (MW PF)	● Managing exclusions ● Managing emotions when conducting the intervention: developing confidence
4.Peer facilitator training and supervision (PF)	● Length ● Organization ● Content ● Obstacles dealt with ● Supervisor recommendations
5.What a valuable tool to have: benefits of the interactive community map (MW HV PF)	● Visual tool ● Means of empowerment
**Overarching theme: *Going where women are***	● Locations – going to women
	● Managing Mistrust
	● Acknowledging women’s needs

In this theme peer facilitators also discussed the emotions and/or competing demands they had to deal with when delivering the intervention, including the need to develop their own confidence, and disappointment if women were allocated to the control arm and their input was not required. Random allocation could on occasion mean no women were allocated to intervention on some sessions. However, a peer facilitator had to be available as this was unpredictable, but if all women fell in the control arm the peer facilitator would not be utilised that day.

“It was just a bit disappointing like, for ourselves to get here, to sit here for hours and then no one turns up” (PF8)

- 4. Training and supervision
  “I preferred to get it done, rather than have half a day, half a day, half a day isn’t it, it’s easier, isn’t it?” (PF4)
  This theme related to peer facilitator perspectives on training and supervision. Peer facilitator supervisors commented very positively on the duration, content and process of the training, which was provided over four short days to facilitate peer facilitators child caring responsibilities:

“They made it dead interactive. There was loads of up and down and different stuff wasn’t there, yeah, I really enjoyed it.” (PF5)

Supervision was also well received.

“I thought that was enough time weren’t it? And there was enough time for everyone to have a talk in that time as well” (PF5) ( Note. Speaking about the supervision sessions)

- 5. ‘What a valuable tool to have’—benefits of the interactive community map
  “The visual thing … the map was the visual thing that was needed, because I find a lot of our parents are visual so they writing’s down but if they can see it in front of them, they can see, yeah, that’s great” (PF8).
  Most peer facilitators, midwives and health visitors viewed the community map as an invaluable visual tool:

### Preliminary effectiveness information

#### Primary outcome: community service use

Uptake of any community service assessed via the Client Services Receipt Inventory (CSRI) during pregnancy was generally low, with over half of all participants (55%) indicating that they made no use of community services between TIME 1 and TIME 2.

There was a trend for intervention group participants to have a higher mean number of antenatal contacts with community services (M = 5.00, SD = 12.75) than control group participants (M = 2.48, SD = 5.24) but this difference ( t = -0.95 *p* = 0.344) was not significant. Similar trends were seen in the postnatal period, with the intervention group having a greater number of postnatal contacts (M = 17.92, SD = 18.33) than control group participants (M = 12.38, SD = 10.10) but again this was not significantly different (t = -1.30, *p* = 0.199). There were no significant differences between intervention and control groups in terms of the number of types of community services accessed or the mean duration of contacts with community services antenatally or postnatally.

#### Secondary outcomes: psychological wellbeing measures across antenatal and postnatal phases

Two-way mixed ANOVAs were carried out on scores for the psychological wellbeing measures that were administered at all three timepoints (HADS, WEMWBS). Table 4 shows means and standard deviations across time points.

**Table 4:** Means and standard deviations in brackets for time points and study conditions for anxiety, depression and wellbeing

	**Time 1**		**Time 2**		**Time 3**	
	**Intervention**	**Control**	**Intervention**	**Control**	**Intervention**	**Control**
**Anxiety (HADS)**	**7.17(3.42)**	**4.70(2.39)**	**5.75(3.34)**	**4.45(2.52)**	**5.92(4.15)**	**4.80(4.07)**
**Depression (HADS)**	**3.43(2.63)**	**2.10(1.89)**	**3.91(2.76)**	**3.35(2.21)**	**4.87(3.08)**	**4.30(3.94)**
**Wellbeing (WEMWBS)**	**52.21(8.82)**	**55.86(8.94)**	**53.21(8.34)**	**57.43(6.29)**	**53.46(10.68)**	**54.93(10.68)**

*Anxiety (HADS)*: There was no statistically significant interaction between study condition and time point on anxiety scores [*F*(1.67,70.06) = 0.89, *p* = 0.399];. There were no significant changes in anxiety scores observed over the three time points across intervention and control group participants [*F*(1.67,70.06) = 1.21, *p* = 0.299]; or any differences in anxiety scores between intervention and control group participants [*F*(1,42) = 3.99, *p* = 0.052].

*Depression (HADS)*: There was no statistically significant interaction between study condition and time point on depression scores [*F*(2,82) = 0.426, *p* = 0.655]. A main effect of time was observed [*F*(2,82) = 7.152*p* = 0.001] with depression scores increasing over the course of the study across intervention and control groups. There were no significant differences in depression scores *between* intervention and control group participants.[*F*(1,41) = 1.54, *p* = 0.221].

*Wellbeing (WEMWBS:)*There was no statistically significant interaction between study condition and time point on wellbeing scores [*F*(1.67,71.68) = 0.08, *p* = 0.891]. There were no significant changes in wellbeing scores observed over the three time points across intervention and control group participants [*F*(1.67,70.06) = 0.53,*p* = 0.561] and no significant differences in wellbeing scores between intervention and control group participants [*F*(1,43) = 2.67, *p* = 0.11]. There were no significant differences between the intervention and control groups at Time 3 in terms of total parenting stress (t = -0.664, *p* = 0.509), parental distress (t = -0.931, *p* = 0.355), difficult child (t = 0.086, *p* = 0.932) or parent–child dysfunctional relationship (t = 0.169, *p* = 0.867) subscale scores on the PSI-SF or on the MORS in terms of warmth (t = 0.781, *p* = 0.438), or invasiveness subscale scores (t = -0.148, *p* = 0.883).

## Discussion

This study shows that the three elements (peer facilitation, identification of needs with signposting and ‘*If–Then’* planning) can be meaningfully combined and adapted for use in the perinatal context. This brief intervention was acceptable to women in the perinatal period in areas of very high deprivation. Importantly, women reported that this legitimised their rights in the perinatal phase to take their own needs into account, as expressed in the theme of *Bringing Mum into focus*.

Women receiving the intervention valued the personal interaction with the peer facilitator and most said the timing ‘as early as possible in pregnancy’ was appropriate. However, this raises issues for the design of a future definitive randomised controlled trial, as a proportion of participants became ineligible after the first intervention session. Many women felt that the postnatal intervention needed to be later than 6 weeks after birth. They also requested a phone option instead of a face-to-face meeting with the peer facilitators.

Midwives and health visitors believed strongly in the underpinning philosophy of individualised and tailored care through identification of women’s individual needs and steps supporting them to facilitate implementation of their wishes. They felt this could be easily integrated into services and indeed wanted all the women they saw to have access to this without exclusion. Midwives had some reservations about adding to the time of the already lengthy first booking appointment but were aware of the complexities of alternatives. The multidisciplinary stake holder and service user group agreed that, on balance, this was the best timing.

Peer facilitators could be trained and retained in their role. The training package was well received and positively evaluated in terms of the length, content, and utility in preparing the peers for delivery of the intervention. They suggested that further time spent on navigating the interactive community map during training would be beneficial.

Peer facilitators were able to provide the intervention programme with high levels of fidelity. However, they needed support to negotiate some of the emotional challenges such as the unpredictability of uptake and use when involved in research, and developing confidence in the provision of a novel intervention.

The external validity of perinatal intervention studies is often compromised by biased participation from more educated and socially affluent samples [29]. However, this study demonstrates that women living in areas with the highest level of deprivation can be recruited to this intervention and retained in a randomised research trial design.

A variety of studies have examined barriers to engagement with health and community services in areas of relative deprivation. Downe et al. [30] examined barriers to antenatal care for marginalised women in high-income countries and identified issues about quality of care provided, personal resources and social support, trustworthiness and the perceived lack of mutual respect. Cleland et al. [31] investigating barriers to engagement with community prevention services for coronary heart disease in areas of relative deprivation, showed that perceived low personal relevance of offered interventions can act as a barrier to utilisation. It appears that the PeARS programme directly addresses some of these barriers. Women viewed the intervention as highly relevant and the positive inter-personal qualities of the peer were highly valued. The emphasis on *Going where women are* physically, socially and emotionally and *Bringing the mum into focus* address the key issues often impeding engagement. For example, maternity care providers frequently spoke about the mistrust they encountered from the women toward both antenatal and postnatal care provisions. Maternity care staff, particularly midwives, highlighted that a key element in this intervention was provision by a peer, which might optimise uptake, in comparison to provision by a less trusted professional.

Peer support during the perinatal period has been shown to ‘have a number of interrelated positive impacts on the emotional wellbeing of mothers’ [32]. In itself it can be a valuable perinatal mental health intervention [4] and may also interact with the uptake of the uptake of more community based resources. This current study utilised less intensive peer facilitation to make use of existing community resources rather than provide active ongoing peer support. Despite this, women still appreciated their peer facilitators. They highlighted the importance of continuity of the person providing the support, to allow for trusting relationships to be developed and to limit repetition of information. The wish for continuity of midwife and the potential benefits to perinatal women has already been well documented [33].

The study was not powered to detect differences but to explore feasibility. Nevertheless, there was a trend for intervention group women to have higher mean contacts with community services during both the antenatal and postnatal periods. However anxiety, depression and wellbeing in this small sample showed no indication of difference. Attendance at postnatal peer facilitation meetings was low, and interviews suggested they needed to be held later in the postnatal period. However, in those attending, 95% reported *If–Then* plan completion. This suggests that the intervention may be of particular relevance and resonance for specific subgroups who relate to this sort of intervention.

In summary, the PeARS programme is feasible to deliver reliably and is acceptable to both women and service providers. It now requires large scale systematic evaluation to determine efficacy and cost effectiveness. The PeARS community map is essentially a signposting feature of the intervention. Whilst signposting is seen as an important feature of social prescribing now embraced by health services it is notable that there is limited empirical supporting evidence perinatally or in any population groups [34]. Only 35 (just over 40%), of the social prescribing and/or signposting interventions identified by Thomson et al. [35] have been evaluated, and of these just 5% were evaluated using robust quantitative designs such as randomised control trials (RCTs). The authors recommend that all social prescribing interventions should have a minimum of pre/post evaluation, and, where appropriate, RCTs should be considered. The data presented in this paper offer the basis for such an RCT to evaluate both clinical and the cost-effectiveness of this novel intervention.

## Limitations

The views and outcomes of participants are of necessity those who have engaged in the study and may not be representative of those who declined participation. The study excluded women who did not book with community midwife led care. Participating women and maternity care providers emphasised the importance of inclusion for all pregnant women. In addition, use of community resources pre-pregnancy was not measured, and therefore we do not know if there was initial group equivalence on this measure at baseline. Exclusion criteria should be re-examined, to assess if a future full trial should become more inclusive to offer a universal provision.

## Conclusion

The PeARS study feasibility tested and piloted an innovative low-intensity community-based intervention delivered by peers to pregnant and postnatal women living in an area of very high deprivation. The results suggest that the programme appears to overcome some of the barriers to engagement in services associated with social deprivation. Qualitative information suggests it is acceptable to women and to service providers and it can be integrated into existing provision. Research processes were acceptable and adequate numbers of women agreed to be recruited, and stayed with the programme. Preliminary results concerning engagement in local services and implementation of individualised plans are encouraging. There were indications that a subset of women may find the approach particularly helpful and it is likely that no ‘one size fits all’ and that women may need the opportunity of a range of ways of accessing support made available to them.

## Supplementary Information

**Additional file 1**: Topic guide for 6month postnatal follow up.

**Additional file 2**: Intervention fidelity monitoring checklist.

## Data Availability

Qualitative data extracts are presented in the article to support the findings. The original transcripts are not available to the public as they may contain information that could compromise the confidentiality and anonymity of the participants. However Professor Slade, the corresponding author (pauline.slade@liverpool.ac.uk) may respond to reasonable requests for anonymised data.

## Abbreviations

AMP: Mental Health in Primary Care
CSRI: Client Service Receipt Inventory
DC: Difficult Child
HADS: Hospital Anxiety and Depression Scale
IMD: Index of Multiple Deprivation
PCDI: Parent–Child Dysfunctional Interaction
PD: Parental Distress
PeARS: Perinatal Access to Resources and Support
PSI: Parenting Stress Index
MORS: Mother Object Relations Scale
RCT: Randomized Control Trials
WEMWBS: Warwick Edinburgh Mental Wellbeing Scale

## Footnotes

1 Children’s centres in the UK are financed by local councils to provide a range of services for families from conception to age 5.
