# Supplementary material for: Facilitating Perinatal Access to Resources and Support (PeARS): a feasibility study with external pilot of a novel intervention
Source: BMC Pregnancy Childbirth. 2021 Nov 12;21:769. doi: 10.1186/s12884-021-04112-w (PMC8588611; doi:10.1186/s12884-021-04112-w)
Supplement: Supplementary file 1 — Additional file 1: Topic guide for 6month postnatal follow up. [file 12884_2021_4112_MOESM1_ESM.docx]

**Additional File 1**

**TOPIC GUIDE FOR 6MONTH POSTNATAL FOLLOWUP**

**(INTERVENTION GROUP) ( ITALICS INDICATE FOLLOWUP PROMPTS)**

1. Did you receive information about the study before we saw you at the booking in

appointment with your midwife at the children’s centre?

• *If yes, what was good/not so good about it?*

2. At the booking in appointment were you given the opportunity to ask any questions

you may have had before deciding to take part in the study?

• *Were any questions/concerns you had answered?*

3. How did you find completing the questionnaires?

• *What was good/not so good (can you give me some examples?)*

4. The researcher gave you a booklet with details of all the local services on offer. Did

you use this booklet?

• *If yes, what for?*

5. What did you like best/least about being involved in the study and why?

6. What do you think about the idea of meeting with a female worker to discuss any

support needs you might have during pregnancy and make an individualized plan on

how to access these services?

7. When in pregnancy do you think it would be best to do this?

• *Early, middle or late?*

8. What do you think would help women to take up this service?

9. What do you think could get in the way?

10. How would you feel about the same female worker contacting you to see how you

are getting on with your plan?

10th October 2016 Version 1.0

11. What are your thoughts about having this service also available for women after

they have had the baby?

12. How would you feel about having a female worker available on the phone in

pregnancy and postnatally in case your needs change?

• *How useful do you think this would be?*

13. Is there anything we could have do differently (do you think) to improve the study in

terms of the individualized planning?

14. Is there anything else you’d like to add before we finish the interview?

**(CONTROL GROUP) ( ITALICS INDICATE FOLLOWUP PROMPTS)**

1. Did you receive information about the study before we saw you at the booking in

appointment with your midwife at the children’s centre?

• *If yes, what was good/not so good about it?*

2. At the booking in appointment were you given the opportunity to ask any questions

you may have had before deciding to take part in the study?

• *Were any questions/concerns you had answered?*

3. How did you find completing the questionnaires?

• *What was good/not so good (can you give me some examples?)*

4. The researcher gave you a booklet with details of all the local services on offer. Did

you use this booklet?

• *If yes, what for?*

5. What did you like best/least about being involved in the study and why?

6. What do you think about the idea of meeting with a female worker to discuss any

support needs you might have during pregnancy and make an individualized plan on

how to access these services?

7. When in pregnancy do you think it would be best to do this?

• *Early, middle or late?*

8. What do you think would help women to take up this service?

9. What do you think could get in the way?

10. How would you feel about the same female worker contacting you to see how you

are getting on with your plan?

10th October 2016 Version 1.0

11. What are your thoughts about having this service also available for women after

they have had the baby?

12. How would you feel about having a female worker available on the phone in

pregnancy and postnatally in case your needs change?

• *How useful do you think this would be?*

13. Is there anything we could have do differently (do you think) to improve the study in

terms of the individualized planning?

14. Is there anything else you’d like to add before we finish the interview?
