# Supplementary material for: Facilitating Perinatal Access to Resources and Support (PeARS): a feasibility study with external pilot of a novel intervention
Source: BMC Pregnancy Childbirth. 2021 Nov 12;21:769. doi: 10.1186/s12884-021-04112-w (PMC8588611; doi:10.1186/s12884-021-04112-w)
Supplement: Supplementary file 2 — Additional file 2: Intervention fidelity monitoring checklist. [file 12884_2021_4112_MOESM2_ESM.docx]

**Appendix : Intervention Fidelity Monitoring Checklist**

Has the peer facilitator completed the following… (*please tick*) YES/NO

**Introduced the session to the participant?**

**Identified a relevant support need**

**(using the *what if* questions as prompts if necessary)?**

**Signposted the participant to an appropriate resource based on her preference using**

**the community resource directory?**

**Helped the participant develop an effective if-then plan that meets key criteria**

**(RELEVANT, SPECIFIC, REALISTIC, LINK SITUATION TO BEHAVIOUR, MEMORABLE)?**

**Helped the participant to record her if-then plan?**

**Asked the participant to rehearse (x3) her if-then plan?**

**Identified a potential barrier to completing the if-then plan?**

**Helped the participant to develop an if-then coping plan?**

**Helped the participant to record her if-then coping plan?**

**Asked the participant to rehearse (x3) her if-then coping plan?**

**Given the participant positive reinforcement?**

**Given the participant the relevant community resource booklet(s)?**

**Notified the participant about follow-up telephone contact?**

**Checked the participant’s contact number(s) and given them their number?**

**Used good non-verbal communication skills (active listening, body language etc.)**

**throughout the session?**

**Used good verbal communication skills (appropriate questions, reflecting etc.) to**

**engage the participant and elicit relevant information?**

**Remained warm and approachable throughout the session?**

**Worked within the boundaries of the Peer Facilitator role?**

**Please note any areas requiring further input from research team: ……………………………………**

**…………………………………………………………………………………………………………………………………………..**

**…………………………………………………………………………………………………………………………………………..**

**Any further comments on peer facilitator’s delivery of the intervention: ………………………….**

**…………………………………………………………………………………………………………………………………………..**

**…………………………………………………………………………………………………………………………………………..**

**Feedback given to peer facilitator: YES / NO**
